# Cervical trans-spinal direct current stimulation: a modelling-experimental approach

**DOI:** 10.1186/s12984-019-0589-6

**Published:** 2019-10-25

**Authors:** Sofia Rita Fernandes, Mariana Pereira, Ricardo Salvador, Pedro Cavaleiro Miranda, Mamede de Carvalho

**Affiliations:** 1 Instituto de Fisiologia, Instituto de Medicina Molecular, Faculdade de Medicina, Universidade de Lisboa, Avenida Professor Egas Moniz, 1649-028 Lisbon, Portugal; 2 Instituto de Biofísica e Engenharia Biomédica, Faculdade de Ciências, Universidade de Lisboa, Campo Grande, 1749-016 Lisbon, Portugal; 3 Neuroelectrics, Avinguda Tibidabo, 47 bis, 08035 Barcelona, Spain; 4 Departamento de Neurociências e Saúde Mental, Hospital de Santa Maria - Centro Hospitalar Lisboa Norte, Avenida Professor Egas Moniz, 1649-035 Lisbon, Portugal

**Keywords:** Direct current stimulation, Spinal cord, Cervical, Neuromodulation, Computational modeling

## Abstract

**Background:**

Trans-spinal direct current stimulation (tsDCS) is a non-invasive technique with promising neuromodulatory effects on spinal cord (SC) circuitry. Computational studies are essential to guide effective tsDCS protocols for specific clinical applications. This study aims to combine modelling and experimental studies to determine the electrode montage that maximizes electric field (E-field) delivery during cervical tsDCS.

**Methods:**

Current and E-field distributions in the cervical SC were predicted for four electrode montages in a human realistic model using computational methods. A double-blind crossover and randomized exploratory study was conducted using the montage that maximized E-field delivery. tsDCS was applied for 15 min in 10 healthy subjects (anodal, cathodal, sham, with polarity assigned to the cervical electrode), with a current intensity of 2.5 mA, resulting in a total current charge density delivery of 90 mC/cm^2^. Upper limb motor (transcranial magnetic stimulation) and sensory evoked potentials (MEP, SEP), M-waves, H-reflex and F-wave responses were analysed. Central and peripheral conduction times were determined using MEP. Repeated measures ANOVA and Friedman test were used for statistical analysis (significance level α = 0.05).

**Results:**

All montages presented higher current density and E-field magnitudes in the cervical SC region between the electrodes. However, electrodes at C3 and T3 spinous processes (C3-T3) originated the highest E-field magnitude (0.50 V/m). Using C3-T3 montage we observed significant changes in N9 SEP latency (*p* = 0.006), but significance did not persist in pairwise comparisons (sham-anodal: *p* = 0.022; sham-cathodal: *p* = 0.619; anodal-cathodal: *p* = 0.018; α = 0.017, Bonferroni corrected). MEP latency and central motor conduction time (CMCT) modified significantly on stimulation (*p* = 0.007 and *p* = 0.015, respectively). In addition, pairwise comparisons confirmed significant differences between sham and cathodal conditions after Bonferroni correction for MEP latency (sham-anodal: *p* = 0.868; sham-cathodal: *p* = 0.011; anodal-cathodal: *p* = 0.023) and CMCT (sham-anodal: *p* = 0.929; sham-cathodal: *p* = 0.010; anodal-cathodal: *p* = 0.034).

**Conclusions:**

Computational models predicted higher E-field delivery in the cervical SC for the C3-T3 montage. Polarity-dependent effects in motor responses were reported using this montage consistent with spinal motor modulation. tsDCS experimental protocol designs should be guided by modelling studies to improve effectiveness.

## Background

Upper limb sensorimotor innervation arises mostly from the cervical region of the spinal cord (SC). Several clinical conditions, such as upper limb weakness, sensory deficit or pain, can be associated with cervical spinal circuitry dysfunctions.

Trans-spinal direct current stimulation (tsDCS) has recently emerged as a non-invasive technique with promising neuromodulatory effects on spinal circuitry related to motor and sensory responses of the upper and lower limbs [7, 8]. tsDCS has a similar approach as transcranial direct current stimulation (tDCS), a non-invasive brain stimulation method for modulating cortical excitability [28]. It applies a constant low intensity electric current through surface electrodes. However, tsDCS and tDCS differ in their principles of application due to the significant heterogeneity of their target tissues. Whereas anodal tDCS produces facilitation in cortical motor responses [28], exploratory tsDCS studies in humans report a variety of polarity-dependent effects in spinal motor responses when stimulating the cervical SC: facilitation of motor responses of abductor digiti minimi (ADM) and abductor policis brevis (APB) muscles was observed during cathodal tsDCS applied over C7 spinous process (s.p.), with the anode over the right deltoid (rD) muscle (C7-rD montage, [4]); increased amplitude of motor evoked potentials (MEP) of the flexor carpi radialis (FCR) was observed independently of the polarity of the electrodes, placed at C7 s.p. and cervicomental angle (C7-CMA montage; [22]). Cervical tsDCS was also observed to increase corticophrenic pathway excitability, considering a C4-CMA montage: increased diaphragmatic MEP amplitude was observed independently of C4 polarity and tidal volume was increased with the cathode placed at C4 [25].

tsDCS neuromodulatory effects may result from local variations of the current density and electric field (E-field) along neurons, resulting in specific polarizing effects in the transmembrane potential, with axon terminals identified as the dominant cellular target [34, 37]. These variations are affected by various stimulation parameters such as electrode placement and geometry, or injected current intensity and polarity, just as in tDCS [15, 20, 41].

Computational studies using realistic human models based on MRI are essential tools to predict the electrode montages and stimulation parameters that maximize current delivery and E-field distribution in a specific clinical target [11, 20, 24, 30]. There are few modelling studies published on tsDCS delivery on human lumbar and thoracic SC regions; these studies predict maximum E-field magnitude between the electrodes with a stronger longitudinal component in the spinal canal [15, 16, 20, 31]. Modelling predictions seem to explain physiological measures obtained during experimental conditions in cervical tsDCS, using MRI-based rat models or simple geometric human models of the cervical SC: diverse electrode montages resulted in different current and E-field distributions, corresponding to different physiological outcomes [12, 49].

The aim of this study is to present a modelling work considering three electrode montages previously explored in the studies cited above, and a longitudinal montage with two electrodes over the SC, since this is thought to result in less variability in experimental results, compared to anterior-posterior montages, as suggested in Dongés et al. [13]. Next, we proceed to present the results of an experimental study carried out in healthy human volunteers using this longitudinal electrode montage, to address neuromodulatory effects on motor and sensory pathways of the upper limb.

## Methods

### Modelling study

#### Human and electrode models design

A realistic human model was designed based on the 34 years-old male Duke of the Virtual Population Family [9]. Fifteen tissues were considered (Table 1), with no distinction between white matter (WM) and grey matter (GM) in brainstem and cerebellum. The spinal-GM was artificially designed considering SC anatomy [43] and measurements from the Visible Human Data Set (National Library of Medicine, NLM, Visible Human Project®, www.nlm.nih.gov/research/visible/visible_human.html). The full model was truncated at the level of the thighs and above the elbows, to shorten computational time.

**Table 1:** Isotropic electrical conductivities of tissues in the human trunk model

*Tissue*	*σ (S/m)*	*Reference*
Skin	0.435	[17]
Fat	0.040	[18]
Muscle	0.355 (av)	[39]
Lungs	0.046 (av)	[39]
Heart	0.535 (av)	[47],
		[18]
Viscera (liver, pancreas, stomach, small and large intestine, air)	0.123 (av)	[47],
		[29],
		[18]
Vertebrae/Bone	0.006	[18]
Intervertebral disks	0.200	[18]
Dura mater	0.030	[46]
CSF	1.790	[3]
Brainstem / Spinal roots	0.154	[18]
Cerebellum	0.290 (av)	[10], [18]
Spinal-WM	0.143	[18]
Spinal-GM	0.333	[18]

**Fig. 1:**
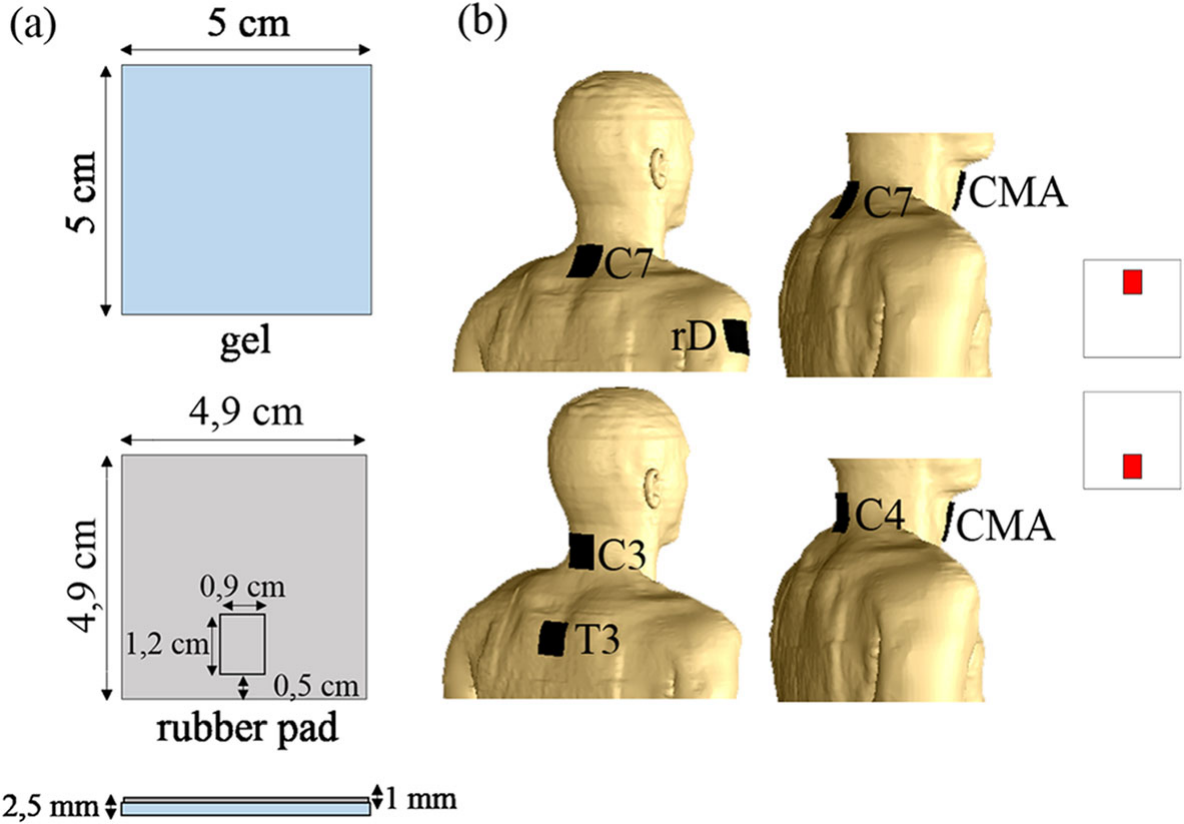
Electrode settings: (**a**) gel, rubber pad and connector dimensions; (**b**) montages considered in the study, with illustration of connector configuration on the right

Electrodes were designed as gel and rubber rectangular prism layers, with a metallic rectangular connector on the rubber’s surface, considering the Fiab Spa silicone electrode (Vicchio, Italy, http://www.fiab.it) used in the experimental study (Fig. 1a). The three electrodes montages applied in the aforementioned cervical tsDCS studies were modelled first to address the E-field distribution and magnitude variability with electrode position (Fig. 1b): C7-rD [4]; C7-CMA [12, 13, 22]; C4-CMA [25]. Considering the E-field characteristics predicted from the three previous montages, a new electrode placement on C3 and T3 s.p. (C3-T3) was simulated, to test if higher E-fields could be observed in cervical segments related with upper limb function (C4 to T1 spinal segments) compared to C7-rD and CMA montages. C3-T3 was already simulated in a preliminary study from our group, with promising results [14].

Surface meshes were optimized and assembled, and volume meshing was performed with the 3-MATIC module from MIMICS (MIMICS software, v16), resulting in 2.0 × 10^7^ tetrahedral elements for the entire human model with electrodes.

#### Electrical properties of tissue and electrode materials

Tissues and materials were assumed to be purely resistive. Isotropic electrical conductivity values were assigned to each tissue in the model after a literature review on DC electrical tissue properties and compiled in Table 1. Cerebellum conductivity resulted from a volume-weighted average of WM and GM conductivities, using volume estimates from Damasceno et al. [10].

Anisotropic conductivity tensors were calculated for muscle and spinal-WM, according to the method described in Fernandes et al. [15], considering different transverse and longitudinal muscle conductivities (σ_trans_ = 0.043 S/m, σ_long_ = 0.667 S/m, [39]), and using the volume constraint from Wolters [48] for the spinal-WM. Conductivity matrices were assigned for each mesh node using a Matlab script (v2015b, www.mathworks.com) and conductivity tensors were interpolated for each volume element using COMSOL Multiphysics (4.3b, www.comsol.com).

A conductivity of 4 S/m [23] was assigned to the gel, and rubber pad conductivity was measured to be 44 ± 1 S/m, according to the method described in Fernandes et al. [15]. Gel and rubber conductivities were considered as isotropic.

#### Electric field calculation and analysis

E-field calculations were performed with COMSOL Multiphysics using the finite element method. Current intensity was set to 2.5 mA and boundary conditions were applied according to Miranda et al. [24], considering electrode connectors as isopotential surfaces. The cervical electrode was considered as the cathode and the other electrode as the anode. Reversing polarity would invert the direction of the E-field but would not affect its magnitude [38]. Four simulations were performed (one per montage), with 2.7 × 10^7^ degrees of freedom and solution time of about 150 min per simulation on a computer with 2 quad-core Intel® Xeon® processors clocked at 3.2 GHz and 48 GB of RAM.

Modelling studies in tDCS reported volume-average E-field values larger than 0.15 V/m over the hand knob, when reproducing clinical settings with observed neuromodulatory effects [24, 26, 41]. tsDCS neuromodulation will be assumed if the average E-field exceeds this value in the SC.

E-field components were defined as 3 orthogonal vectors oriented as: caudal-rostral and tangent to SC axis (${\overrightarrow{E}}_{long}$); ventral-dorsal (${\overrightarrow{E}}_{vd}$); right-left (${\overrightarrow{E}}_{rl}$). Average values in spinal-WM and GM were determined in 1 mm thick axial slices along the z-axis.

### Experimental study

Higher E-field magnitude in the cervical SC was predicted for C3-T3 montage, therefore we proceeded to a randomized double-blinded observational study in humans to address sensorimotor responses using this montage.

#### Subjects

The experimental study was performed on 10 healthy right-handed volunteers (6 women), 22 to 40 years old (mean age 31), recruited from students and staff of the Faculdade de Medicina da Universidade de Lisboa (FMUL). Exclusion criteria applied were: neurologic or psychiatric disease, diabetes, use of biomedical devices. The protocol was approved by the local Ethics Board of the academic centre and all subjects gave informed consent.

#### Materials and recording conditions

Skin surface was cleaned with an abrasive gel and a solution of ethyl alcohol 96% before placing superficial recording and stimulating electrodes. Subjects remained seated in a comfortable armchair. Upper limb was positioned with shoulder in slight abduction (60°), elbow semi-flexed (110°), and forearm pronated and supported by the arm of the chair [33]. The lower limb was also tested to address possible distal effects of the stimulation. It was positioned with 120° hip flexion, 160° knee flexion and 110° plantar flexion of the ankle [33]. The tested muscles were the abductor digiti minimi (ADM) in the right upper limb and the abductor hallux (AH) in the right lower right limb, both were placed in neutral position and relaxed. Each session lasted 90 min approximately. The temperature of the room was kept constant (23–25 °C).

#### tsDCS experimental protocol

DC stimulation was applied by a commercially available stimulator (BrainSTIM©, SEM, Bologne, Italy) connected to a pair of silicone rubber pad square electrodes 1 mm thick and 25 cm^2^ area (5 × 5 cm^2^) from Fiab Spa (Vicchio, Italy, www.fiab.it). Electrodes were placed above C3 and T3 s.p.. Conductive gel (Signa Gel®, Parker, USA) was applied between the electrodes and the skin to reduce and stabilize contact impedance (< 5kΩ) during stimulation [32]. Direct current of 2.5 mA was applied during 900 s (30 s ramp-up before tsDCS and 30 s ramped-down to 0 mA after tsDCS), with current and charge density of 0.1 mA/cm^2^ and 90 mC/cm^2^, respectively. There are no established safety limits for current density delivery during tsDCS, however, the values indicated above are two orders of magnitude below the threshold limit of 14.3 mA/cm^2^ (143 A/m^2^) determined by Liebetanz et al. [21], for tDCS-induced brain tissue damage. Also, all subjects tolerated well the experimental setting, with no reported burning or itching sensations. Anodal and cathodal conditions were distinguished only by current polarity, referred to the cervical electrode. Sham tsDCS was delivered during 900 s with intensity 0 mA, using active ramp stimulation for 30 s before and after this period, to subjects blind to stimulation condition.

Subjects underwent three sessions of tsDCS, followed by recordings. Session conditions (anodal/cathodal/sham) were randomized with subject and evaluator (MdeC) blinded to the current condition used in each session. Sessions were performed at intervals of at least 1 week in each subject to avoid carry-on effects [27]. Adverse effects, such as burning, itching and pain sensations, were monitored in all subjects.

#### Sensory responses

Electrical bipolar stimulation of the right median nerve at wrist was applied to evoke spinal and cortical somatosensory EPs (SEPs). Two sets of 500 stimuli (0.2 ms pulse width), with an intensity able to induce a slight muscle contraction were applied at 2 Hz, and recordings were performed with Ag/AgCl surface electrodes (impedance < 5 kΩ), according to the guidelines for SEPs assessment in the upper extremity from the American Clinical Neurophysiology Society (ACNS) [2]. The two sets of recordings were applied to improve SEPs measurements average, as recommended by the ACNS [2]. The recording electrodes were placed on: ipsilateral Erb’s point (referred to the contralateral Erb’s point); C7 s.p (referred to the anterior neck); 1 cm behind C3 and C4 on the scalp (referred to Fz). Amplitude, peak-latency for N9, N13, N18, N20 and P22 and interpeak-latencies were recorded.

#### Motor responses

Neurophysiological motor responses were recorded with EMG equipment (Keypoint© Dantec-Natus) using conventional electrodes (Alpine BioMed®, Denmark, Ref 9013 L0203). Skin impedance under EMG electrodes was kept < 10 kΩ, according to the International Federation of Clinical Neurophysiology guidelines (IFCN [36];).

##### Motor evoked potentials (MEP)

Transcranial magnetic stimulation (TMS) was performed using a conventional circular coil (14 cm diameter coil, Medtronic MagPro©, MagVenture, Denmark), centered on the vertex, to induce maximum electric current near the outer edge of the coil in the motor cortex. Motor responses were recorded from right ADM and AH muscles using a belly-tendon montage. Resting motor threshold was estimated as the lowest intensity needed to elicit motor responses ≥50 μV in at least 5 of 10 stimuli [35, 36]. MEPs were obtained by stimulating the cortex 20% above threshold with 30 s interstimulus interval. We considered mean MEP amplitude and latency from 10 consecutive MEPs. Peripheral and central motor conduction times (PMCT and CMCT, respectively) were calculated for upper limb responses as in Kimura et al. [19]: PMCT = ½(M_latency_ + F_minimum latency_ – 1); CMCT = MEP_latency_ – PMCT.

##### Cortical silent period (CSP)

We stimulated the motor area (stimulus frequency < 0.1 Hz) as described above while the subject made a moderate contraction of the right ADM to determine hand CSP (determined from stimulus artefact to electrical signal return with amplitude > 0.1 mV). We measured the mean CSP duration from 10 sweeps. This method has been shown to be reliable on blind rater evaluation [5].

##### F-waves and H-reflex responses

These responses were measured in 5 subjects to infer lower motor neuron (LMN) excitability following tsDCS. F-waves were recorded from right ADM following supramaximal stimulation of the ulnar nerve at wrist (20 stimuli, 1 Hz, filter setting 20–10,000 Hz). We evaluated mean peak-to-peak amplitude, negative-peak area, minimum, maximum and mean latency, chronodispersion and persistence before and after each intervention.

H-reflex was recorded using a bipolar montage over the flexor carpi radialis (FCR) muscle, and by delivering 1 ms rectangular stimuli in the median nerve at elbow. The frequency of stimulation was 0.5 Hz to avoid post-activation depression [33]. Applied current intensity was progressively increased to obtain H-reflex in steps of 0.2 mA, until eliciting the maximal M-wave. H-reflex threshold, maximum H-reflex amplitude and minimum latency, and H:M ratio were evaluated.

#### Statistical analysis

Experimental sessions were compared considering the factor condition (sham, cathodal, anodal) using repeated-measures ANOVA. The Greenhouse-Geisser correction was applied when Mauchly’s test of sphericity was significant. The Friedman test (with Wilcoxon test for paired comparisons) was applied for non-normal data. A *p*-value < 0.05 was set as significant following Bonferroni correction for multiple comparisons. Data is presented as mean ± standard deviation (STD). Calculations were performed with IBM SPSS, version 25.

## Results

### Modelling study

This section presents the main results for current density and E-field distribution in the spinal cord and adjacent regions. Additional information can be found in Additional file 1.

#### Current density distribution in the human model

**Fig. 2:**
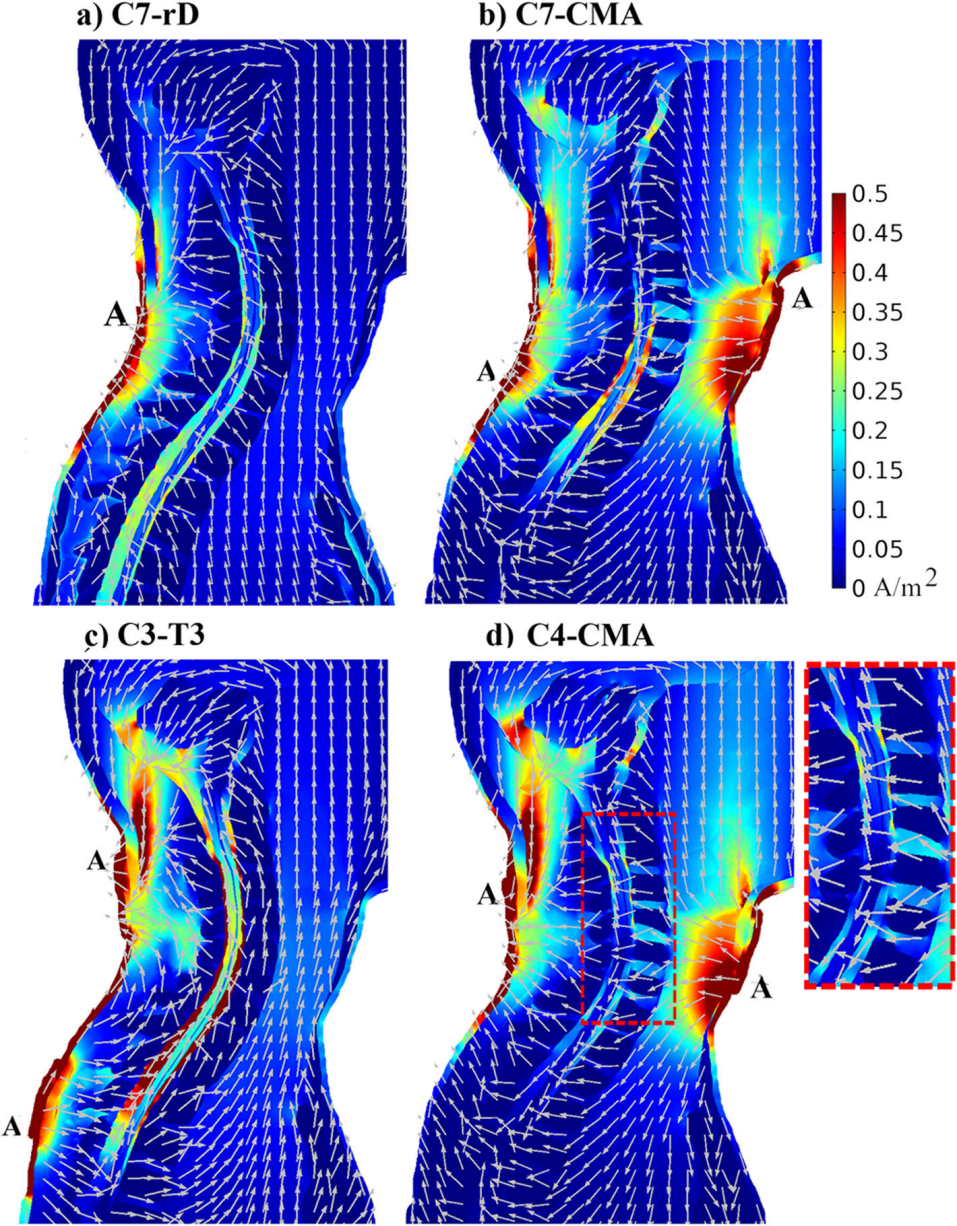
Current density magnitude distribution in a sagittal slice of the upper thoracic and neck regions for each electrode montage, with the current direction represented by grey arrows of the same length: **a**) C7-rD, **b**) C7-CMA; **c**) C3-T3; **d**) C4-CMA. Letter “A” marks the active connector position in each electrode. The magnitude colour scale is on the top right and is the same for all montages. The inset on the right of C4-CMA shows the reversal in current density direction inside the spinal canal

Current flows longitudinally from anode to cathode in the spinal canal, due to anatomical shape and high CSF conductivity, decreasing from skin to spinal-GM by two orders of magnitude (Fig. 2). Current orientation changes in C4-CMA at the level of the edge of CMA active connector (C6/C7 vertebral region; Fig. 2d, inset). This may result from the combination of anatomical characteristics, such as different electrical conductivities, and electrode position. Cerebellum and brainstem present local current maxima, which are larger in montages with electrodes placed at a higher cervical position (C4-CMA; C3-T3). These local maxima may be originated by current flow originated in the highly conductive muscle and CSF tissues.

#### E-field distribution in the spinal cord and adjacent regions

**Fig. 3:**
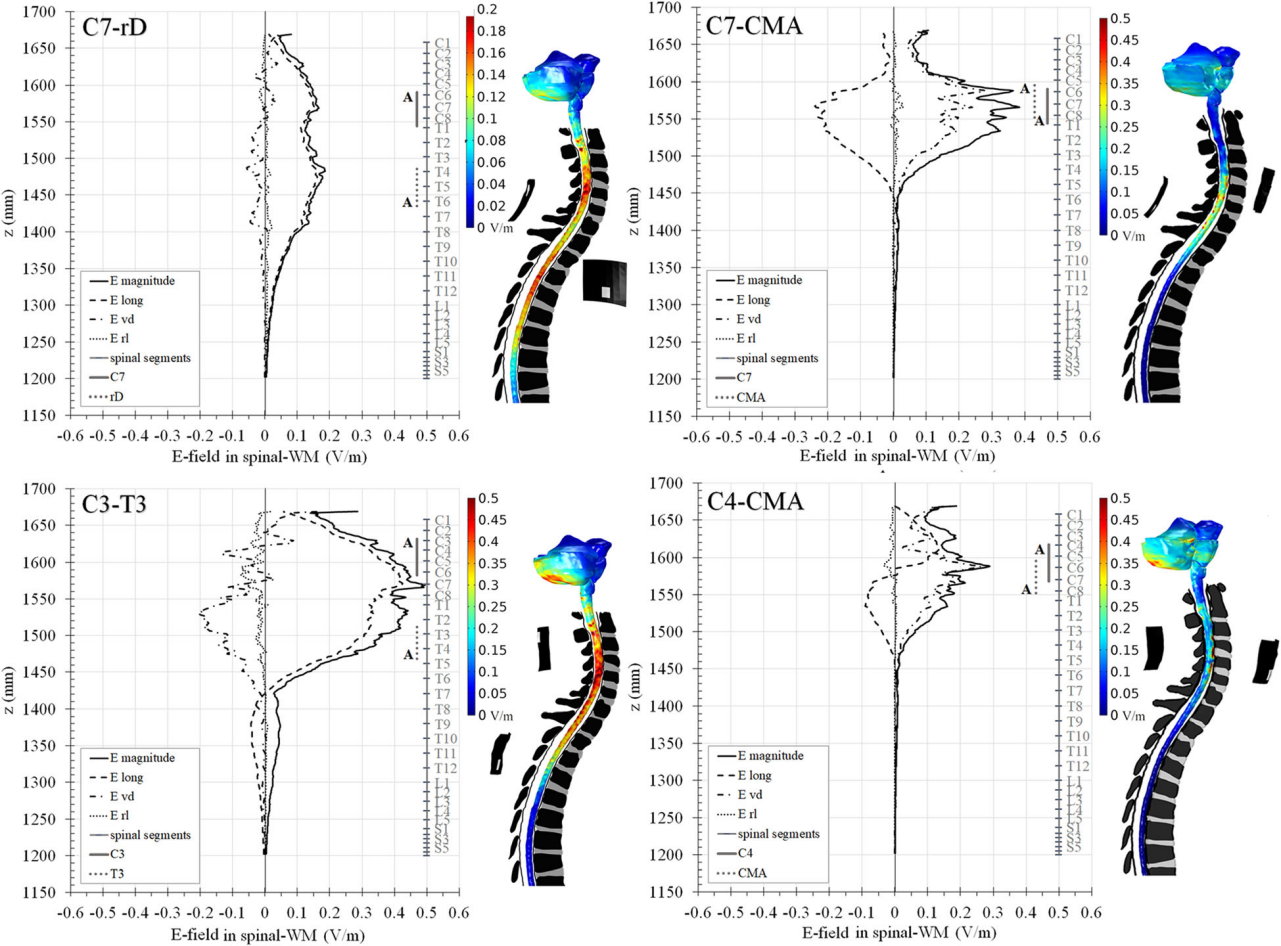
Average magnitude of the E-field and average amplitude of its components in the spinal-WM in all montages along the z axis. Position of spinal segments is marked on the grey vertical bar, electrodes are represented by vertical bars and active connectors are marked with letter “A”. Volume plots of the E-field magnitude in cervico-thoracic spinal-WM, brainstem and cerebellum are represented at the right of the average distribution in each montage, with the corresponding colour scales

Figure 3 presents profiles of the E-field magnitude and of its components averaged over the spinal-WM along the caudal-rostral direction. Similar profiles were observed for the spinal-GM. Considering 0.15 V/m as the minimum E-field value for neuromodulation, different montages address different clinical targets, as shown in Table 2. All montages may modulate upper limb functions, higher cervical montages (C4-CMA, C3-T3) may also address mechanical-related respiratory functions. C3-T3 presents the highest magnitudes, reaching 0.40 and 0.50 V/m in C6-C7 segments of the spinal-GM and WM, respectively. E_long_ makes the largest contribution to the E-field magnitude in C7-rD and C3-T3, as E_vd_ and E_rl_ have much lower values, reflecting the longitudinal nature of the field. CMA montages have comparable E_long_ and E_vd_ components in the spinal-WM, due to the posterior-anterior electrodes location. E_long_ reverses direction along the SC in C4-CMA as in the current density profile (Fig. 3, lower right).

**Table 2:** Spinal segments with E-field magnitude above 0.15 V/m and related body regions

Montages	C7-rD	C7-CMA	C4-CMA	C3-T3
*Spinal-WM*	C6-C7, T2-T5	C4-T1	C2-T1	C1-T5
*Spinal-GM*	C6-C7, T2-T5	C5-T1	–	C1-T4
*Body regions*	Upper limb Upper thoracic region	Neck, Shoulder, Upper limb, Wrist, Hand	Neck, Diaphragm, Shoulder, Upper limb, Wrist, Hand	Neck, Diaphragm, Shoulder, Upper limb, Wrist, Hand Upper thoracic region

The E-field value also exceeds 0.15 V/m in posterior regions of the brainstem and cerebellum, especially in higher cervical montages (C3-T3 and C4-CMA), which is consistent with the current density magnitude observed in these regions (Fig. 2b and c); this may indicate that neuromodulation of these regions can also occur during tsDCS application.

E-field localized maxima or hotspots appear in the same regions of the spinal-GM and WM in all montages. Moderate to strong coefficients of determination were found for inverse function fits between E-field magnitude (E_mag_) and CSF volume (V_CSF_) distributions in local spinal regions for each montage (V_CSF_ x E_mag_^a^ = constant (a > 0), R^2^ = 0.5–0.9). CSF narrowing in the spinal canal may be the main anatomical feature causing E-field hotspots. However, disks and vertebrae bony edges protrusions may cause CSF narrowing and originate hotspots indirectly.

**Fig. 4:**
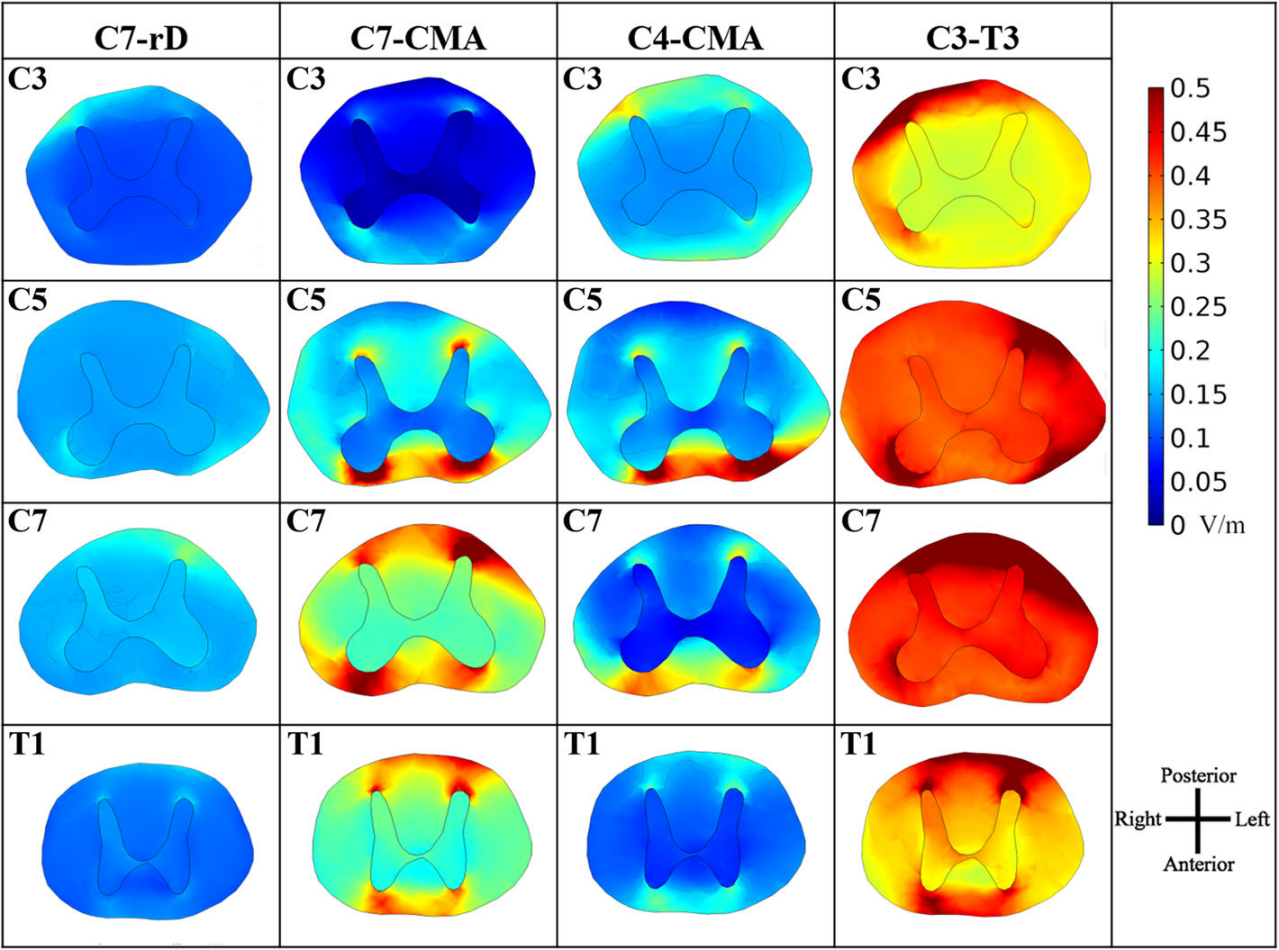
Axial SC slices in selected cervical and T1 segments near maximum E-field peaks. Colour scale and image orientation are represented on the right

Axial slices of the E-field magnitude in spinal segments near maxima present an almost constant magnitude in the GM, with local maxima near WM/GM interface, especially at dorsal and ventral horns, where most collateral fibres enter or leave the GM (Fig. 4). C3 and C7 segments have different maxima positions in spinal-WM when comparing CMA montages with C7-rD and C3-T3: local maxima appear also in the anterior regions in CMA montages. This indicates an influence on electrode position: the CMA montage originates a higher ventral-dorsal component that originates higher E-field in the anterior regions of the spinal-WM segments near or between the electrodes.

### Experimental study

Only the most significant changes are reported in this section. Additional information on experimental measurements, statistics and comparison tests results is reported in Additional file 1.

#### Sensory pathways

**Fig. 5:**
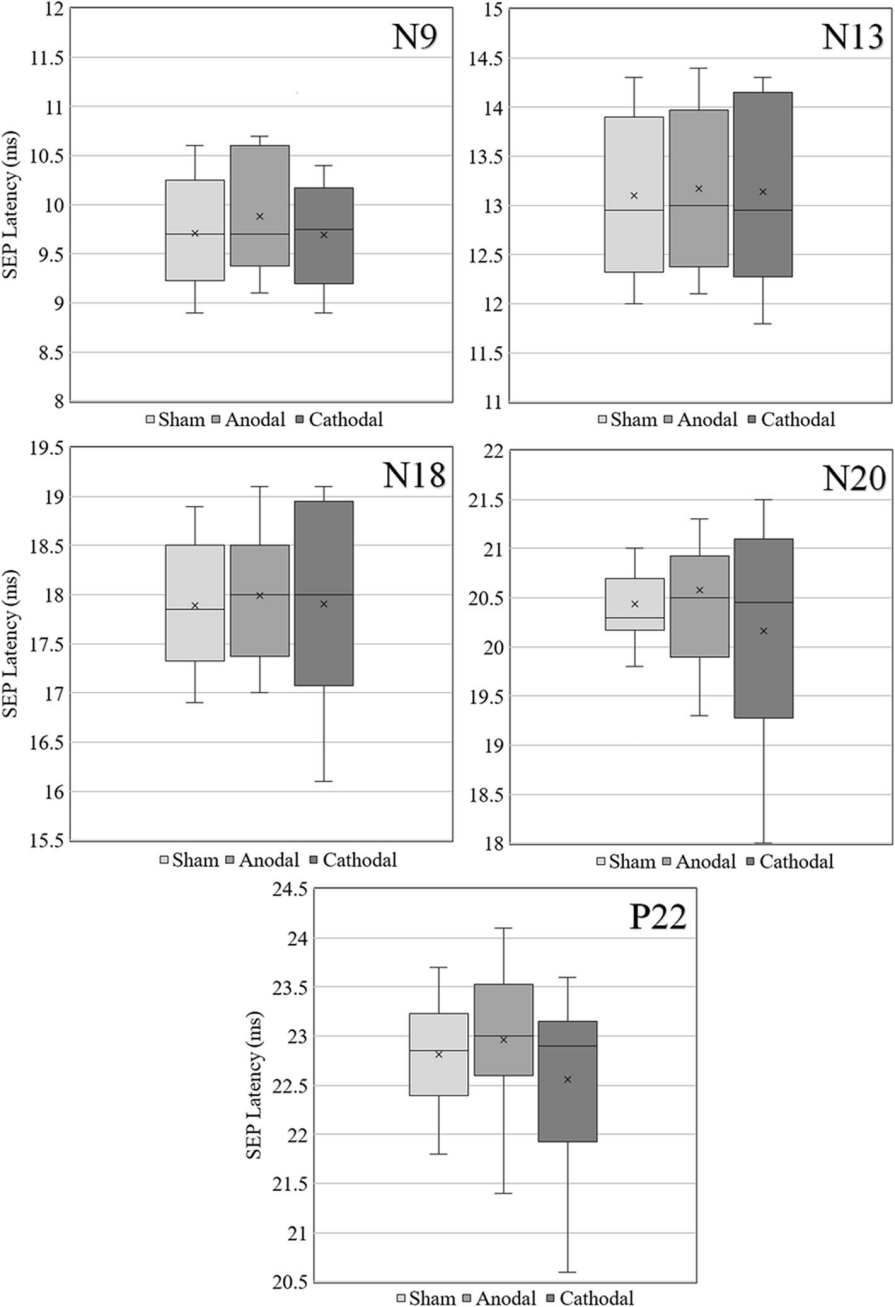
Boxplots of N9, N13, N18, N20 and P22 SEP’s latency for sham, anodal and cathodal conditions

N9, N13, N18, N20 and P22 amplitudes and peak and interpeak latencies were measured immediately after each stimulation condition (sham, cathodal, anodal). SEPs amplitudes and interpeak latencies did not present statistically significant differences. SEPs mean peak latencies presented a tendency to increase after anodal tsDCS (Fig. 5). N9 SEP values attained statistical significance in multiple comparisons between conditions (F(2, 18) = 6.797, *p* = 0.006), however this difference was not maintained after pairwise comparisons (sham-anodal: *p* = 0.022; sham-cathodal: *p* = 0.619; anodal-cathodal: *p* = 0.018; Bonferroni corrected: α = 0.05/3 = 0.017; Fig. 5). No polarity-dependent neuromodulation on upper limb sensory responses can be ascertained from the results presented.

#### Motor pathways

**Fig. 6:**
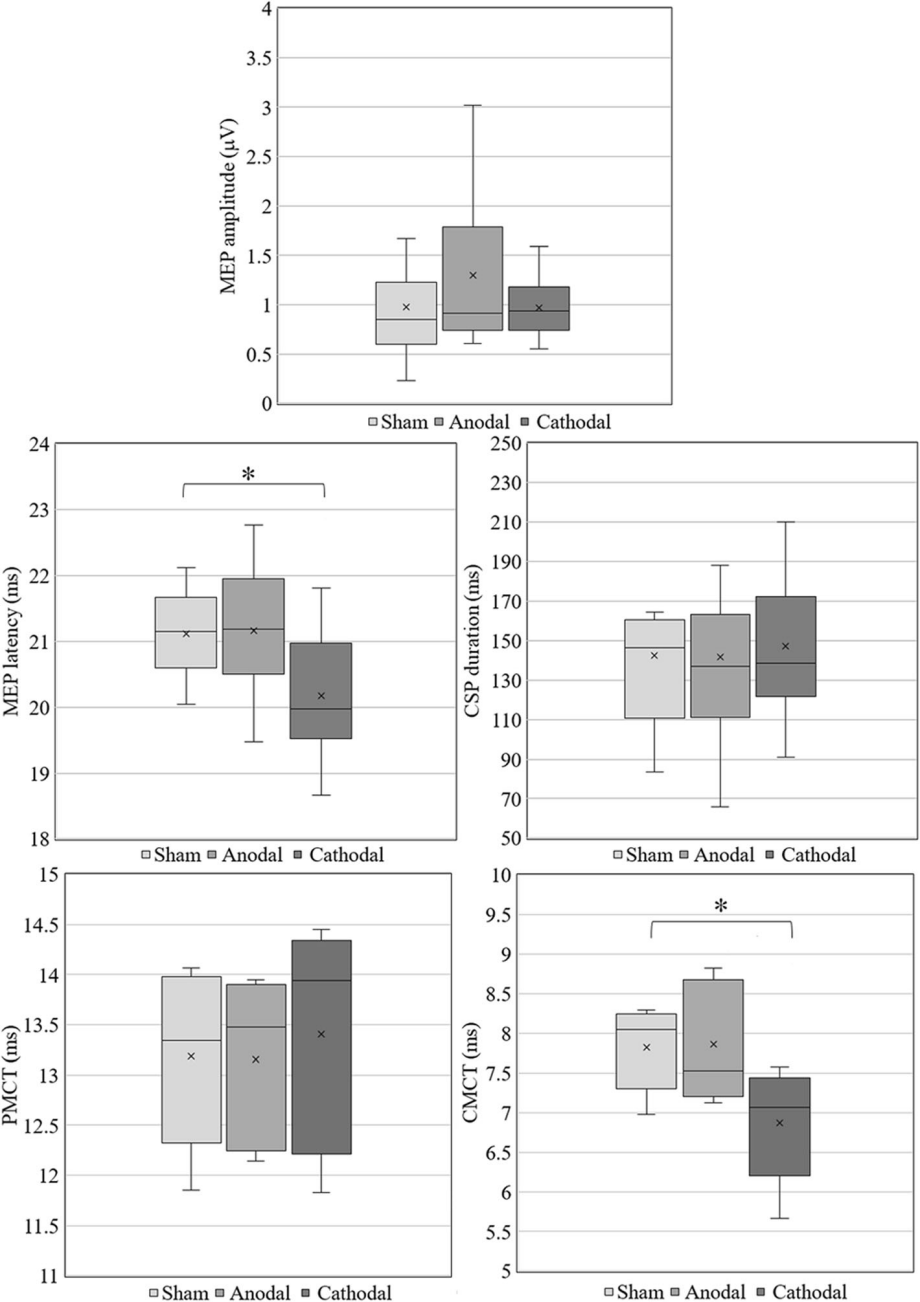
Boxplots of upper limb MEP amplitude and latency, CSP duration, PMCT and CMCT for sham, anodal and cathodal conditions. Statistically significant differences between conditions (sham-cathodal) are marked by * (*p* < 0.05/3, Bonferroni corrected) in MEP latency and CMCT

CSP duration and MEP responses (amplitude, area and latency) were measured in the upper limb after sham, anodal and cathodal conditions (Fig. 6). MEP latency recorded in ADM was the only measurement with a significant change (F(2, 18) = 3.139, *p* = 0.007, Table 3). Bonferroni corrected pairwise comparisons revealed a statistically significant difference between cathodal and sham conditions (sham-anodal: *p* = 0.868; sham-cathodal: *p* = 0.011; anodal-cathodal: *p* = 0.023), with cathodal tsDCS resulting in a shorter mean latency. There were no significant changes from multiple comparisons for MEP amplitude (F(2, 18) = 1.303, *p* = 0.296), MEP area (F(2, 18) = 1.324, *p* = 0.291) and CSP duration (F(2, 18) = 0.184, *p* = 0.834; Table 3). Distal effects of tsDCS were addressed with lower limb MEP responses, however these did not present significant changes in amplitude, area and latency (*p* > 0.05; Table 3).

**Table 3:** Neurophysiological motor responses for upper and lower limb (ADM: abductor digiti minimi muscle; AH: abductor hallux muscle; CMCT: central motor conduction time; CSP: cortical silent period; MEP: motor evoked potential; PMCT: peripheral motor conduction time; SEP: somatosensory evoked potential)

Neurophysiological parameters		Mean ± STD			repeated-measures ANOVA results			
		Sham	Anodal	Cathodal	F	df	df error	*p* value
upper limb MEP (ADM)	Amplitude (μV)	1.0 ± 0.6	1.3 ± 0.8	1.0 ± 0.3	1.303	2	18	0.296
	Area (mA)	1.6 ± 1.4	2.1 ± 1.4	1.5 ± 0.6	1.324	2	18	0.291
	Latency (ms)	21.1 ± 0.6	21.1 ± 1.0	20.2 ± 1.0	3.139	2	18	0.007^a^
lower limb MEP (AH)	Amplitude (μV)	1.4 ± 0.9	1.4 ± 0.7	1.4 ± 0.8	0.009	2	18	0.991
	Area (mA)	2.5 ± 2.1	2.5 ± 1.5	2.4 ± 1.7	0.025	2	18	0.976
	Latency (ms)	37.5 ± 2.2	38.2 ± 2.3	37.6 ± 2.1	1.184	2	18	0.329
CSP	Duration (ms)	143 ± 43	142 ± 49	147 ± 36	0.184	2	18	0.834
M-wave	Amplitude (mV)	4.5 ± 1.7	4.7 ± 2.2	3.8 ± 1.7	1.998	2	8	0.198
(median)	Latency (ms)	3.1 ± 0.2	3.1 ± 0.4	3.2 ± 0.3	0.201	2	8	0.822
H-reflex (median)	H_max_ (mV)	0.3 ± 0.2	0.3 ± 0.2	0.5 ± 0.6	1.104	2	8	0.377
	Min Lat (ms)	21.2 ± 1.6	21.0 ± 2.1	22.3 ± 2.6	1.284	2	8	0.328
	H:M	0.08 ± 0.05	0.06 ± 0.03	0.12 ± 0.10	1.601	2	8	0.260
	Threshold (mA)	3.4 ± 1.4	3.8 ± 1.1	3.5 ± 1.3	0.582	2	8	0.581
M-wave	Amplitude (mV)	16.4 ± 3.3	16.1 ± 3.6	16.0 ± 3.3	0.562	2	8	0.591
(ulnar)	Latency (ms)	2.3 ± 0.1	2.2 ± 0.2	2.4 ± 0.3	4.072	2	8	0.060
F-wave (ulnar)	Amplitude (μV)	206 ± 163	203 ± 155	214 ± 145	0.310	2	8	0.742
	Area (μA)	903 ± 838	794 ± 761	890 ± 683	0.924	2	8	0.436
	Min Lat (ms)	25.1 ± 1.8	25.1 ± 1.7	25.4 ± 2.3	0.454	2	8	0.651
	Mean Lat (ms)	26.8 ± 1.8	27.2 ± 1.9	27.6 ± 1.6	1.530	2	8	0.274
	Max Lat (ms)	29.0 ± 1.9	29.1 ± 2.0	29.8 ± 2.3	1.046	2	8	0.395
	Chronodispersion^c^	3.9 ± 1.4	4.0 ± 1.3	4.4 ± 1.7	0.508^b^	1.041	4.164	0.521
	Persistence	18 ± 2	16 ± 5	18 ± 3	2.344	2	8	0.158
PMCT^d^	Duration (ms)	13.2 ± 0.9	13.2 ± 0.9	13.4 ± 1.1	1.106	2	8	0.377
CMCT^e^	Duration (ms)	7.8 ± 0.5	7.9 ± 0.8	6.9 ± 0.7	7.422	2	8	0.015^a^
	Min Duration (ms)	7.1 ± 0.7	6.7 ± 0.9	6.3 ± 1.0	2.610	2	8	0.134

^a^ Statistically significant differences within subjects (*p* < 0.05)

^b^ Greenhouse-Geisser correction

^c^Chronodispersion (ms) = maximum latency – minimum latency

^d^PMCT = 0.5 x (M-wave mean latency + F-wave minimum latency - 1)

^e^CMCT = 0.5 x (MEP mean latency – PMCT); CMCT minimum = 0.5 x (MEP minimum latency – PMCT)

M-wave, F-wave and H-reflex responses were measured in 5 subjects to address if MEP latency changes could be due to central or peripheral effects of stimulation. There were no statistically significant changes in multiple comparisons between sham, anodal and cathodal conditions for M-wave, H-reflex, H:M ratio and F-wave measurements, as reported in Table 3 (*p* > 0.05 in all comparisons). Combining the results from F-wave and MEP responses, the PMCT and CMCT were determined in the 5 subjects. Only the CMCT presented statistically significant differences in multiple comparisons (F(2, 8) = 7.422, *p* = 0.015, Table 3), a significant reduction was disclosed following cathodal stimulation (sham-anodal: *p* = 0.929; sham-cathodal: *p* = 0.010; anodal-cathodal: *p* = 0.034, α = 0.017, Bonferroni corrected, Fig. 6).

## Discussion

### Current density and E-field predictions from the modelling study

Current density and E-field distributions have a higher longitudinal component in the SC. Longitudinal fields were reported in other modelling studies on thoracic and lumbar tsDCS [15, 16, 20, 31], This longitudinal tendency is due to the cable-like structure of the spine, with a conductive core (spinal cord and CSF) surrounded by an insulating sheath (vertebral column).

All montages present regions with E-field magnitude above 0.15 V/m, which is in line with the observed neuromodulatory effects using C7-rD, C7-CMA and C4-CMA, however these regions are located in different part of the SC (Fig. 3, Table 2), which may account for the differences observed between studies using different montages. Niérat et al. [25] observed increased excitability of the corticophrenic pathway using C4-CMA montage, which is consistent with a larger E-field magnitude predicted in C3-C5 segments, related with the phrenic nerve (Fig. 3). Bocci et al. [4] reported improved upper limb motor recruitment and shortening of peripheral silent period (PSP) after cathodal-tsDCS using C7-rD, which is predicted to induce a stronger field in C6-C7 spinal segments, from where part of the brachial plexus arises. Exploratory studies using C7-CMA montage report different effects, such as MEP amplitude increase without changes in H-reflex [22] or only acute changes in MEP amplitude during combined cathodal-tsDCS and cervicomedullary stimulation [12]. The length of E-field maximum regions also indicates which montages are suitable for wider (C3-T3, C7-rD) or more focal (CMA montages) stimulation.

Local E-field maxima appear mostly near the WM/GM interface at the dorsal and ventral horns (Fig. 4). Previous numerical modelling studies on invasive spinal cord stimulation found that the EF component parallel to fibres (corresponding to E_long_ in SC) have an influence on the transmembrane potential of collateral fibres, originating from the spinal-WM columns, as they bent into the spinal-GM [45]. The same effect was also predicted in transcranial magnetic stimulation (TMS): there may be stimulation of pyramidal tract neurons in the regions where these fibres bend after entering the cortical WM [40]. The neuromodulatory effects that were observed in the exploratory studies on cervical tsDCS mentioned above may be due to the strong E-field variations along the collateral fibres as they bend and pass the WM/GM interface.

The E-field reaches magnitudes above 0.15 V/m in posterior regions of brainstem and cerebellum in higher cervical electrode montages (C4-CMA, C3-T3), thus neuromodulation of vegetative functions can be considered (Fig. 3). Since these tissues were considered homogeneous, a model with distinct WM and GM will allow more accurate predictions of tsDCS effects.

The inverse relations found between E-field magnitude and CSF volume distributions were also observed in thoracic and lumbar tsDCS studies [15, 16]. Individual anatomical variability may originate different tsDCS clinical outcomes, thus subject-specific models should be considered to predict the optimal electrode montages.

### Neuromodulatory effects of C3-T3 montage

C3-T3 resulted in the highest current density and E-field magnitudes, with maxima at C6-C7 spinal segments, where part of the upper limb innervation arises. Longitudinal bipolar montages (both electrodes over the SC) or monopolar montages (one electrode over the target area and the other at a considerable distance) were recommended by Dongés et al. [13] for upper limb function neuromodulation, due to the strong longitudinal component induced. We observed neuromodulatory effects on upper limb sensorimotor responses using C3-T3.

#### Effects on sensory responses

A significant difference was only detected for SEP N9 (F(2, 18) = 6.797, *p* = 0.006), however the significance did not resist pairwise comparisons (Fig. 5). Brachial plexus EP/N9 SEP responses are mainly generated by nerve trunk and roots activity close to the SC [6]. Our model contains nerve exits through vertebral foramina with poor anatomical detail. The E-field distributions presented hotspots near foramina, as seen in animal modelling studies [42]. Spinal roots may contribute to dorsal root ganglia and peripheral nerve excitation, due to a local current focusing caused by the CSF high conductivity near vertebral foramina, supporting our observation.

#### Effects on motor responses

MEP latency in ADM decreased significantly by cathodal stimulation compared with sham condition (Fig. 6). This indicates a polarity-dependent facilitation of motor responses with the cathodal condition, probably caused by increased LMN excitability. In addition, we observed a statistically significant decrease in CMCT by cathodal stimulation, with no changes in PCMT. Both findings are in agreement with previous observations, where a cathodal-dependent LMN facilitation is reported (e.g. [4, 25]). Struijk et al. [44] predicted that cervical epidural stimulation polarity effects vary with spinal dorsal fibres orientation with respect to the E-field: longitudinal and tangential dorsal fibres depolarize near the cathode and radial fibres near the anode. The high E_long_ component predicted in C3-T3 may originate the effects observed on MEP latency, not reported previously in other studies. CSP duration was also addressed in the study to observe if there were effects on inhibitory pathways; however, we did not disclose significant differences. In addition, no effect was observed in lower limb MEP responses (Table 3), suggesting that tsDCS neuromodulation may have only a local effect, without evidence of propagated supra or infraspinal levels effect.

M-waves, H-reflex, F-waves and conduction times were assessed in 5 subjects to address possible tsDCS effects on LMN excitability, but the results were not conclusive. We observed a non-significant increase of the H:M ratio mean values after cathodal condition (Table 3), which might indicate a facilitated MN recruitment by Ia stimulation, also consistent with an increased spinal excitability. One limitation is the small number of tested subjects, in the future this should be repeated in a larger population.

### Considerations on methodology and future research

The number of tissues included in the model was a trade-off between accuracy in field estimates and high-quality volume meshing, to avoid excessive computation time and memory costs. The artificially designed spinal-GM mask is a low-resolution contour that does not represent an accurate change in size across the cervical enlargement. Even so, it was relevant for a more realistic E-field prediction, due to the different WM and GM electric properties.

Future modelling work should address spinal networks to predict the influence of the E-field in neuronal transmembrane potential and understand the variability of observations. Considering the influence of CSF narrowing predicted by the model, it will be important to determine the influence of inter-subject variability in tsDCS outcomes by comparing E-field predictions in different human models.

One of the main limitations of this study is the sample size formed by young subjects. A larger sample with wider age range would be advisable to confirm our experimental findings reported. Also, recording responses during tsDCS and after tsDCS offset could inform on acute and after effects, as observed previously (e.g. [1, 12, 13, 26, 27]). Two further limitations of our study is that we have not explored the modulation of corpus callosum by testing ipsilateral CSP and we did not evaluate phrenic nerve responses to test neuromodulation of C3–5 motor nuclei. Future studies should also address neuromodulation of cerebellum and brainstem circuitry using rostral cervical montages, considering the values of the predicted E-field in those regions.

## Conclusion

This study presents evidence of neuromodulatory effects on sensorimotor spinal pathways using a new cervical tsDCS montage, informed by a computational study based on a realistic human model. Since anatomical features and electrode position influence current and E-field profiles, future tsDCS experimental studies should be optimized by computational models to design effective tsDCS protocols.

## Supplementary information

**Additional file 1.** This file presents plots on the average E-field magnitude in the spinal-GM segments and volume distribution of the E-field magnitude in brainstem and cerebellum, results on correlation fits performed to investigate the influence of anatomical features in local E-field magnitude maxima, and tables summarizing the sensory responses registered during the cervical tsDCS protocol.

## Data Availability

The datasets used and/or analysed during the current study are available from the corresponding author on reasonable request.

## Abbreviations

ACNS: American Clinical Neurophysiology Society
ADM: Abductor digiti minimi muscle
AH: Abductor hallux muscle
ANOVA: Analysis of variance
APB: Abductor policis brevis muscle
CMA: Cervicomental angle
CMCT: Central motor conduction time
CSF: Cerebrospinal fluid
CSP: Cortical silent period
DC: Direct current
E, E-field: Electric field
EMG: Electromyography
FCR: Flexor carpi radialis
FCT: Fundação para a Ciência e Tecnologia
FMUL: Faculdade de Medicina da Universidade de Lisboa
GM: Grey matter
IFCN: International Federation of Clinical Neurophysiology
long: longitudinal
MCTES: Ministério da Ciência, Tecnologia e Ensino Superior
MEP: Motor evoked potential
MN: Motor neuron
MRI: Magnetic resonance imaging
NLM: National Library of Medicine
PMCT: Peripheral motor conduction time
rD: right deltoid
rl: right-left
s.p.: spinous process
SC: spinal cord
SEP: Somatosensory evoked potential
STD: Standard deviation
tDCS: transcranial direct current stimulation
TMS: Transcranial magnetic stimulation
tsDCS: trans-spinal direct current stimulation
vd: ventral-dorsal; WM: white matter
