# Supplementary material for: Cervical trans-spinal direct current stimulation: a modelling-experimental approach
Source: J Neuroeng Rehabil. 2019 Oct 25;16:123. doi: 10.1186/s12984-019-0589-6 (PMC6815068; doi:10.1186/s12984-019-0589-6)
Supplement: Supplementary file 1 — Additional file 1. This file presents plots on the average E-field magnitude in the spinal-GM segments and volume distribution of the E-field magnitude in brainstem and cerebellum, results on correlation fits performed to investigate the influence of anatomical features in local E-field magnitude maxima, and tables summarizing the sensory responses registered during the cervical tsDCS protocol. [file 12984_2019_589_MOESM1_ESM.pdf]

Cervical Trans-Spinal Direct Current Stimulation: a modelling-experimental approach

Supplementary Materials

Average E-field distribution in the spinal-GM

The E-field distribution in the spinal-GM has a similar profile to the spinal-WM (compare figure A below with figure 3 in the main text). This can also be seen in axial slices of spinal segments C2 to T1 (figure B): the E-field magnitudes are similar in both tissues, except mainly near WM/GM interfaces at dorsal and ventral horns. Note that E_{vd} is highest in the spinal-WM when comparing both spinal tissues, with opposite direction in the GM for C7-rD and C3-T3 (figure A). This was not seen in the current density components and it may be due to the spinal curvature, which was used to determine the spinal-WM conductivity tensor.

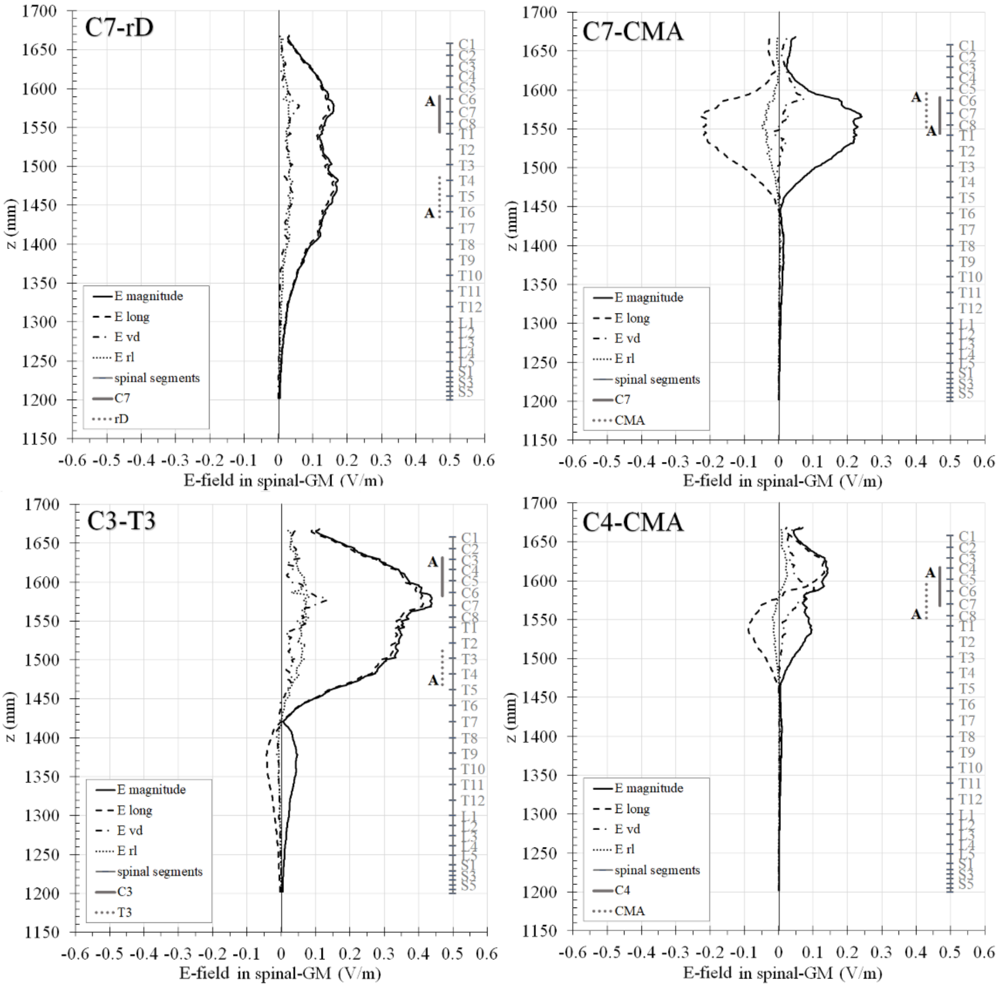

Figure A. Average magnitude of the E-field and average amplitude of its components along the z axis for all montages in spinal-GM. Position of spinal segments are marked on the red vertical bar. Position of electrodes are represented by vertical bars and the position of the active connectors is marked with letter "A".

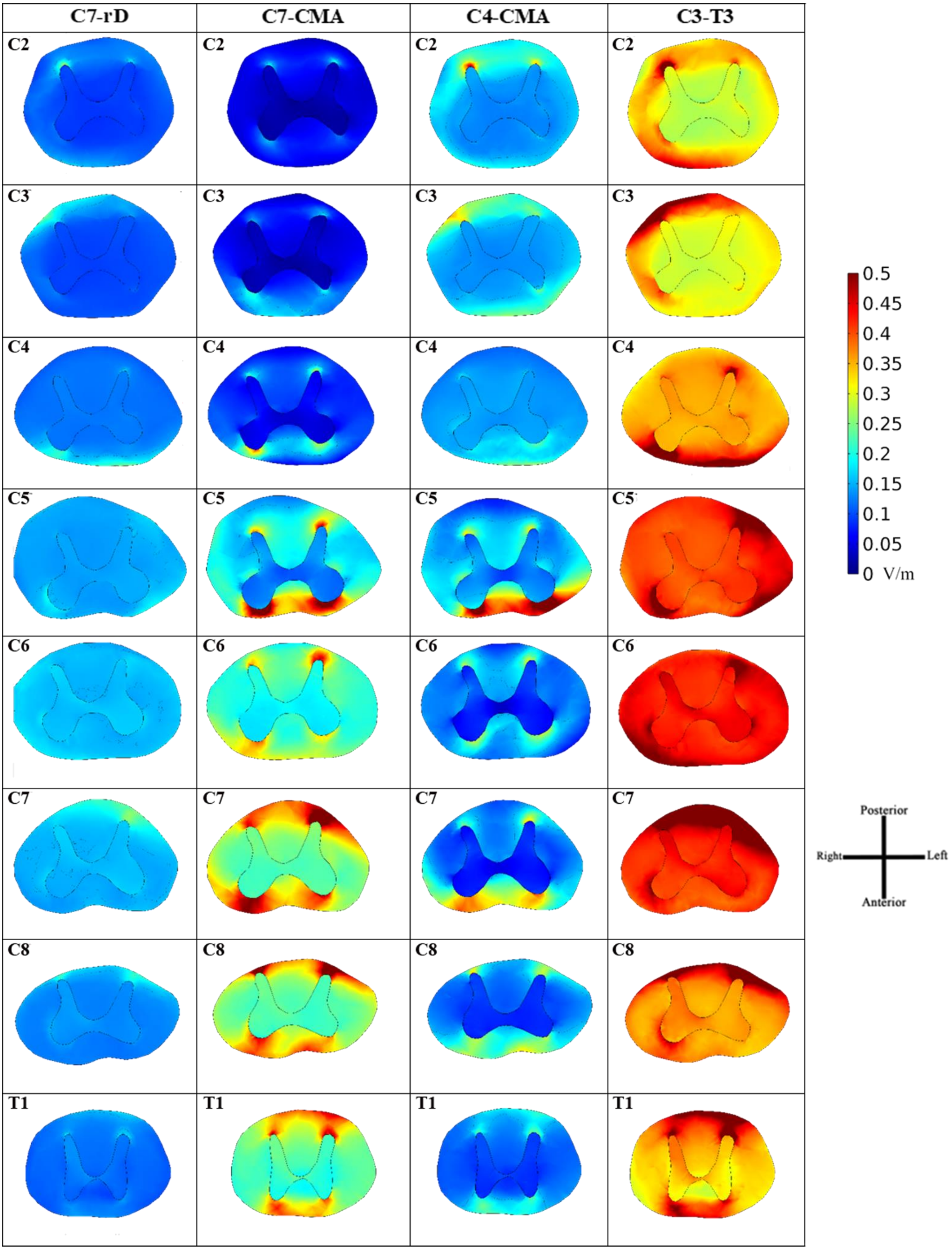

Figure B. Axial SC slices in C2 to T1 segments near maximum E-field peaks. Colour scale and image orientation are represented on the right.

E-field magnitude in brainstem and cerebellum

All montages presented E-field values above 0.15 V/m in the brainstem and cerebellum with regional differences, as seen in figure 3 and shown here in figures C and D. CMA montages will be more suitable for pontine neuromodulation, while C7-rD and C3-T3 may be used for a focal stimulation of posterior medulla oblongata circuitry (figure C). The E-field magnitude is above 0.15 V/m in the interface between the cerebellum and brainstem (cerebellar peduncles), and in the cerebellar posterior hemispheres and vermis, especially for higher cervical montages (C3-T3 and C4-CMA; figure D).

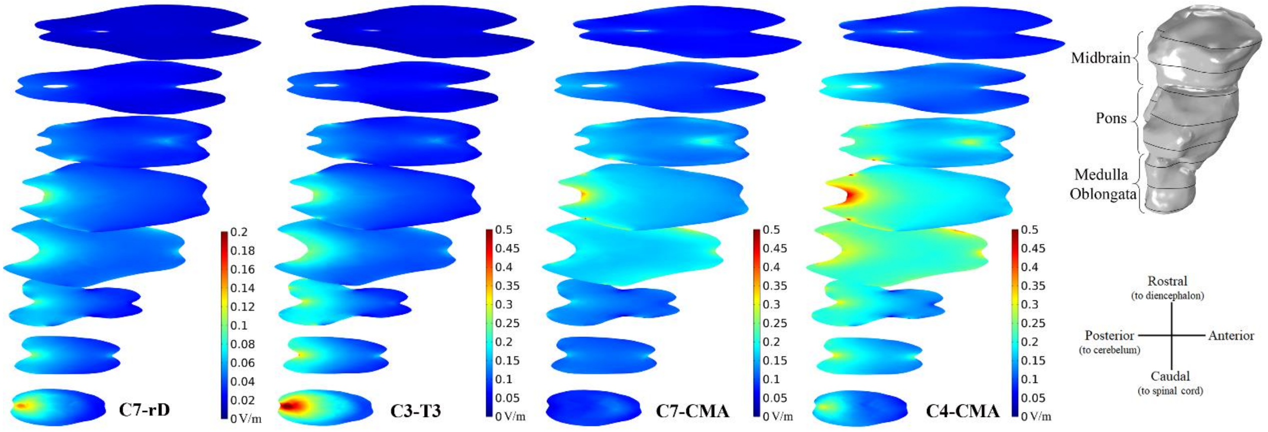

Figure C. Axial slices of the E-field magnitude in the brainstem for all montages with the corresponding colour scale. An inset on the top right corner shows the level of each slice and region in brainstem. Slices orientation is on the bottom right corner.

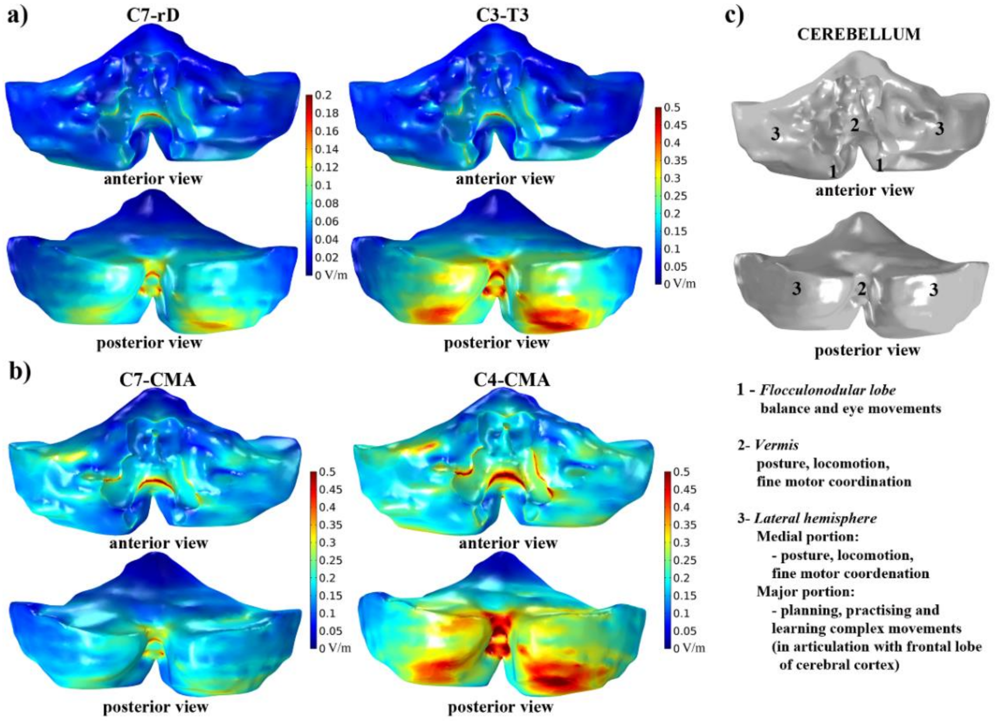

Figure D. E-field magnitude distribution in the cerebellum: a) resulting from C7-rD and C3-T3; b) resulting from CMA montages (C7-CMA; C4-CMA); c) location of the different regions on the cerebellum and specific functions. Anterior and posterior views and the corresponding colour scale are represented for each montage.

Investigating the influence of anatomical features in local E-field magnitude maxima

E-field distributions present maxima peaks in approximately the same positions for all montages, as can be observed in figure 3. These appear to be related with CSF narrowing regions due to vertebrae body or disks intrusions in the spinal canal. Average distributions of the E-field magnitude, CSF volume, disks volume and vertebrae volume in the spinal-WM were compared to address this relation. Inverse correlation fits were found only between average CSF volume and E-field magnitude distributions for two distinct regions of the cervical spinal-WM, with moderate to strong correlations (table A), except for C3-T3 lower cervical region (C6-T1). This is indicative that CSF narrowing may influence E-field maxima location. This may be due to a strong current focusing effect occurring due to the high electrical conductivity of CSF.

Table A. Coefficients of determination for an inverse function* fit between CSF volume (V_{CSF}) and E-field magnitude (E_{mag}) distribution.

**Montage**	**C7-rD**		**C3-T3**		**C7-CMA**		**C4-CMA**	
Spinal segments	**C1-C5**	**C6-T1**	**C1-C5**	**C6-T1**	**C1-C6**	**C7-T1**	**C1-C6**	**C7-T1**
k	$6x10^{-9}$	$2x10^{-10}$	$2x10^{-8}$	$1x10^{-8}$	$4x10^{-8}$	$2x10^{-8}$	$1x10^{-8}$	$2x10^{-8}$
A	1.4	3.3	1.6	2.9	0.6	1.8	1.2	1.2
R^2	0.9	0.7	0.9	0.3	0.7	0.5	0.7	0.7

*Inverse function: V_{CSF} x E_{mag}^a = k

Summary of sensory responses after tsDCS using C3-T3 montage

Tables B and C present mean and STD values of SEPs amplitudes and latencies recorded during the experimental study, and statistics from comparison tests between conditions. SEP amplitudes, peak and interpeak latencies do not present statistically significant changes (sham, anodal, cathodal) except for N9 SEP latency in multiple comparisons.

Table B. SEP amplitude and results from statistical comparisons.

SEP	Amplitude in μV (mean±STD)			repeated-measures ANOVA statistics			
	**Sham**	**Anodal**	**Cathodal**	**F**	**df**	**df error**	**p value**
**N9**	4.46±1.98	4.34±1.68	4.63±2.79	0.133	2	18	0.877
**N13**	1.74±0.35	1.71±0.60	1.86±0.32	0.469	2	18	0.633
**N18**	0.98±0.38	1.11±0.55	1.17±0.69	0.673	2	18	0.523
**N20**	1.84±0.61	2.05±0.48	1.86±0.42	0.545	2	18	0.589
**P22**	1.44±0.71	1.67±1.06	1.48±1.19	1.158	2	18	0.337

Statistically significant differences within subjects considered for $p < 0.05$

Table C. SEP peak and interpeak latencies and results from statistical comparisons.

SEP	Latency in ms (mean±STD)			repeated-measures ANOVA statistics			
	**Sham**	**Anodal**	**Cathodal**	**F**	**df**	**df error**	**p value**
**N9**	9.7±0.6	9.9±0.6	9.7±0.5	6.797	2	18	**0.006**[a]
**N13**	13.1±0.9	13.2±0.8	13.1±0.9	0.400	2	18	0.329
**N18**	17.9±0.7	18.0±0.6	17.9±1.0	0.121	2	18	0.886
**N20**	20.4±0.8	20.6±0.9	20.2±1.1	1.104[b]	1.056[b]	9.503[b]	0.324
**P22**	22.8±0.6	23.0±0.8	22.6±0.9	2.328[b]	1.202[b]	10.815[b]	0.154
**N9-N13**	3.4±0.4	3.3±0.5	3.5±0.5	2.250	2	18	0.134
**N13-N20**	7.3±1.3	7.4±1.1	7.0±1.3	1.201[b]	1.181[b]	10.625[b]	0.309
**N9-N20**	10.7±1.1	10.7±1.0	10.5±1.2	0.538[b]	1.047[b]	9.427[b]	0.489

[a] Statistically significant differences within subjects ($p < 0.05$)

[b] Greenhouse-Geisser correction
